# Ethanol and Methanol Burn Risks in the Home Environment

**DOI:** 10.3390/ijerph15112379

**Published:** 2018-10-26

**Authors:** Torgrim Log, Asgjerd Litlere Moi

**Affiliations:** 1 Department of Fire Safety and HSE Engineering, Glö∂ R&D, Western Norway University of Applied Sciences, 5528 Haugesund, Norway; 2 Department of Health and Caring Sciences, Western Norway University of Applied Sciences, Inndalsveien 28, 5063 Bergen, Norway; Asgjerd.Litlere.Moi@hvl.no; 3 Department of Plastic Surgery and Burn Center, Haukeland University Hospital, Jonas Lies vei 65, 5021 Bergen, Norway

**Keywords:** biofuel fireplaces, biofuel heaters, methanol, ethanol, burn injuries

## Abstract

Biofuel heaters and fireplaces have in recent years been introduced for indoor and outdoor use. Due to their simplicity, they are usually equipped with few or no safety features. Worldwide, incidents resulting in major skin burn injury and long hospitalization periods have occurred when using such biofuel units. The present study analyses the characteristics of the liquids ethanol and methanol to get a scientific background for understanding related accidents. The comparably heavy vapors, especially from ethanol, may generate a pillow of combustible gas in the vicinity of the unit, particularly in quiescent indoor air conditions. It is also revealed that these fuels represent a potential severe risk, since the equilibrium vapor pressures are close to the stoichiometric fuel–air composition at normal room temperatures. Selected incidents were reviewed to understand the mechanisms involved when severe burns were received by the users. It turns out that the most severe incidents were related to refilling operations and included ignition of the fuel container vapor phase. When ignited, the container gas phase expansion propelled burning fuel from the bottle or container onto the user or other persons in the vicinity. Similar incidents involving refilling methanol for chemistry demonstrations and ethanol for endodontic (dentistry) treatment were also studied and it was shown that these accidents followed similar accident mechanisms. It may be concluded that the main contributors to burn risk are the near-stoichiometric vapor pressure of these liquids at room temperature and the close proximity of the fuel container to burning fuel. Research needs and possible technical barriers are suggested to reduce this risk for the future.

## 1. Introduction

Burn injuries are a concern worldwide, with approximately 265,000 burn related deaths every year. Burns are a common injury in all societies and may be a result of hot liquid or hot food spills, contact with hot surfaces, exposure to flames and hot gases, and thermal radiation. In Bangladesh, about 173,000 children under 18 suffer burn-related injuries yearly [1]. In the United States, yearly about half a million patients seek medical treatment at hospital emergency departments in addition to burn injuries treated at clinics, local health centers, and by private institutions [2]. In Norway, the number of fatalities in fires has been reduced significantly during the last 30–40 years. The number of burn victims is, however, according to Smolle et al. [3] not decreasing. Severe burn injuries may result in very long hospitalization periods, and in addition to the physical injury result in major psychological consequences such as body image dissatisfaction, self-harm, post-traumatic stress disorder, anxiety, and depression [4,5,6].

Stimulating the healing of severe burns is difficult. Burn injury treatment therefore has received much research interest [7,8,9]. Improved knowledge about thermal skin injury development, the mechanisms involved, and possible injury-limiting measures are therefore much appreciated. Understanding the situations that may result in burn risks, limiting the likelihood of burns and/or consequences (burn severity), and the promotion of healing processes are therefore necessary measures.

Due to the many burn wounds experienced by the soldiers in the World War II, a systematic approach to studying thermal skin burns was initiated following the war. A series of seminal burn studies was then published in The American Journal of Pathology. These studies included heat transport to and through porcine skin [10], the importance of time and surface temperature in developing cutaneous burns [11], and the pathology and pathogenesis of cutaneous burns on pigs [8]. It was revealed that the degree of burns was dependent on both the temperature and exposure time. A threshold temperature for skin injury was suggested. Several researchers refer to temperature >44 °C for causing burns [12,13]. For hot liquids, others refer to 43 °C for the onset of skin injury [14]. Skin models have also been built for simulating “skin” temperatures during controlled heat flux exposure [15]. It is assumed that the injury develops linearly with time and exponentially with absolute temperature, i.e., an Arrhenius type of injury development.

Human skin pain receptors are located at approximately 0.1 mm depth and the pain temperature threshold is 44.8 °C [16,17]. This is above the assumed threshold temperatures for slow injury development. As burns usually involve much higher basal layer temperatures, the pain signal gives a suitable warning about excessive skin surface heating. In flame exposure scenarios, such as the exposure to the flames from burning biofuel, the skin heating is quite instantaneous, i.e., the damage develops even though the victim is warned of the ongoing process. This is especially dangerous when the victim wears very thin clothing, combustible clothing, or is exposed to burning liquids. Injuries caused by flames, from e.g., kerosene-fueled kitchens/stoves/heaters, represent the most common etiology of burns in adults needing treatment at specialized burn intensive care units [18].

About 20 years ago, bioethanol design fireplaces were introduced in the consumer market. These units typically release combustion products in the indoor air, raising concerns about indoor climate quality [19,20,21]. These units were generally advertised as being ecologically friendly and with a do-it-yourself installation. However, soon after their introduction, patients injured while handling these units showed up at local burn treatment units. One of the first studies regarding these burn injury incidents was an overview of this new recreational fire threat published by Kraemer et al. [22], who warned about an underestimated fire risk among the uninformed customers. Later on, several other research groups also reported that bioethanol design fireplaces were involved in severe burn injuries [23,24,25,26,27]. Many of these incidents were related to refilling operations, and it was reported that burning liquid fuel exposed persons several meters away. Similar incidents have also been reported in dentist offices [28] as well as in educational demonstrations [29] alerting the U.S. Chemical Safety Board (CSB, Washington, DC, USA) about accidents similar to the biofuel incidents [30].

The purpose of the present work is to analyze the hazardous conditions associated with biofuel fireplaces (and heaters) that may result in flame and/or burning fuel exposure to persons operating, and in the vicinity of, these units. Cases reported in the literature are reviewed and analyzed based on the physical and chemical properties of the fuels involved, i.e., methanol and ethanol. Similar incidents involving technically produced methylated liquid fuel products, for educational or professional purposes, are also included in the analysis. Fuel characteristics, such as the lower flammability limit (LFL) and upper flammability limit (UFL), ignition temperatures, and minimum ignition energy (MIE), as well as typical fuel container designs and refueling methods, are analyzed. Typical accident situations as well as fire/explosion risk perception regarding environmentally friendly fuels are discussed. The study is unique in analyzing the whole process, including technical issues regarding the heaters and fuel containers, the clothing when being exposed, and the environmentally friendly, but still very dangerous, fuels involved. 

The motivation for publishing this work is to provide information about the possible accident mechanisms and the victims’ risk perception prior to the incidents as a background for future work for preventing similar accidents. The paper starts with explaining challenges and research on burns and biofuel burns in particular (Section 1). Selected cases from the literature are presented in Section 2. Then the theory of ignition, combustion and heat transfer is presented as well as possible temperature and damage modeling (Section 3). Then, the results based on the previous sections are presented (Section 4) before the results are discussed (Section 5) followed by the conclusions (Section 6). A full analysis as presented in this paper has not been found elsewhere in the literature.

## 2. Physical, Chemical and Fire Related Properties of Methanol and Ethanol

### 2.1. Methanol and Ethanol Vapor Density Relative to Air

The molar masses of methanol and ethanol, $M_{m}$ and $M_{e}$, are 0.03204 kg/mol and 0.04607 g/mol, respectively. The molar mass of dry air, $M_{a}$, is 0.02897 g/mol. The density of gases, or gas mixtures, may be calculated by:

(1) $$\rho =\frac{P\cdot M}{R\cdot T}\;(\mathrm{kg}/m^{3}),$$

where P (101,325 Pa) is the ambient pressure, M (kg/mol) is the molar mass of the actual gas or the average molar mass of the gas mixture, and R (J/molK) is the universal gas constant. Since the density is proportional to the molar mass, Equation (1) demonstrates than the density of methanol, and especially ethanol, is higher than the air. A release of these gases may accumulate at lower levels if there is no mixing by e.g., wind or indoor air circulation. 

### 2.2. Chemical Reaction with Air and Minimum Ignition Energy (MIE)

It is well known that methanol and ethanol are combustible liquids. For simplicity, assuming that the air consists of 21 mol% O_2_ and 79 mol% N_2_ (3.76 mol N_2_ per mol O_2_), the stoichiometric combustion reaction for methanol in air is given by:

CH_3_OH + 1.5 O_2_ + 5.64 N_2_ = CO_2_ + 2 H_2_O + 5.64 N_2_, (2)

and for ethanol it is given by:

C_2_H_5_OH + 3 O_2_ + 11.28 N_2_ = 2 CO_2_ + 3 H_2_O + 11.28 N_2_, (3)

with heat of combustion, $∆H_{c}$, 635 kJ/mol (19.83 MJ/kg) and 1232 kJ/mol (26.78 MJ/kg), respectively [31]. 

Generally, gas–air mixtures ignite most easily at, or close to, the stoichiometric concentrations. For methanol, it may be seen from Equation (1) that this corresponds to 1/8.14 = 12.29% and for ethanol this corresponds to 1/15.28 = 6.54%. 

The heat required for igniting an air methanol or an air ethanol gas mixture at just above stoichiometric conditions, i.e., minimum ignition energy (MIE), is respectively 0.14 mJ and 0.28 mJ. This number is recorded for electrical sparks, but indicates that any burning flame will easily ignite such fuel–air mixtures. It is on the same level as the MIE for familiar flammable hydrocarbons such as propane, gasoline, etc.

Vapors of combustible liquids may also ignite when in contact with hot surfaces. The auto ignition temperature (AIT) is recorded for stoichiometric concentrations. The AITs for methanol and ethanol are 470 °C and 365 °C, respectively [31]. These liquids are therefore not as easily ignited by hot surfaces as long chained hydrocarbon, e.g., diesel with an AIT of 210 °C.

### 2.3. Vapor Pressures and Flammability Limits in Air

**Figure 1:**
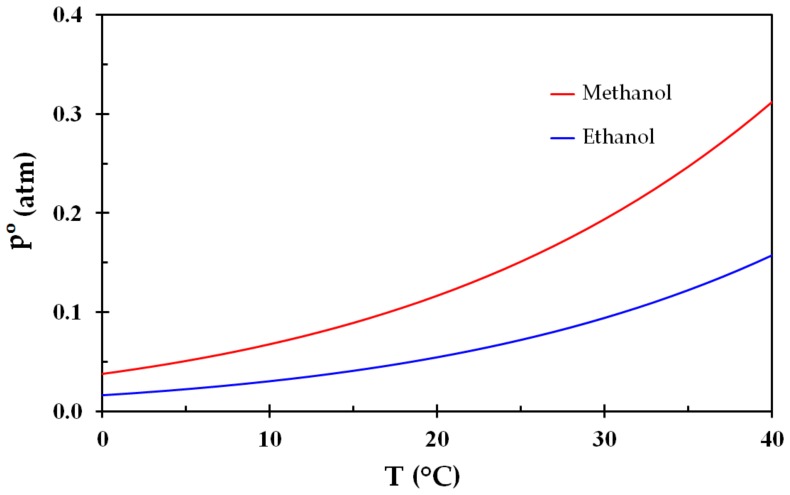
Vapor pressure of methanol and ethanol as a function of temperature (Equation (4)).

The lower explosion limits (LEL) for methanol and ethanol in air are 6.7% and 3.3%, respectively [31]. The respective upper explosion limits are 36% and 19%. The hydrogen bonds between the molecule OH-groups makes these liquids harder to vaporize than their equivalent alkanes, giving heat of vaporization 1.1 MJ/kg and 0.84 MJ/kg, respectively. The vapor pressure of methanol and ethanol as a function of temperature may be expressed by [31]:

(4) $$\log_{10}\left(p^{o}\right)=\left(-\frac{0.2185E}{T}\right)+F,$$

where $p^{o}$ (torr) is the equilibrium vapor pressure. For methanol, the values of the constants are: *E* = 8978.8 K and F = 8.6398, while for ethanol the constants are: E = 9673.9 K and F = 8.8274. The vapor pressure for these liquids is shown in Figure 1. 

**Figure 2:**
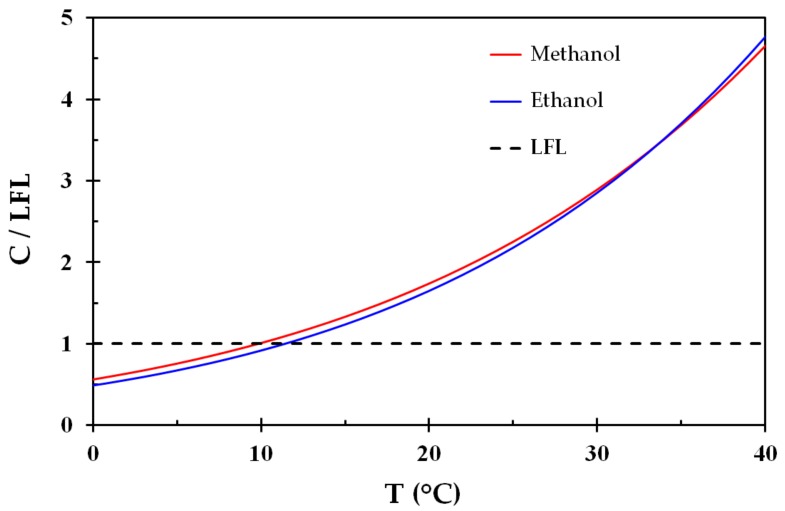
Vapor pressure (concentration, C) normalized by the respective lower flammability limit concentration (LFL) for methanol and ethanol as a function of temperature.

The vapor pressure normalized by the LEL is shown in Figure 2, i.e., where the LEL is represented by the dashed black line. Despite the differences in vapor pressure (Figure 1), these liquids display very similar behavior regarding flammability as a function of temperature. It may be seen from Figure 2 that an air vapor phase at temperatures from about 10–12 °C in equilibrium with any one of these liquids is highly flammable. This is reflected by flash points at around 10–12 °C. 

It should be noted that bioethanol products sold to the consumers also contains 5–10% isopropanol and 1–5% butanone to prevent inadvertent consumption. Isopropanol, i.e., CH_3_CH(OH)CH_3_, has a flash point of 10 °C, i.e., very similar to methanol. Butanone, also known as methyl ethyl ketone (MEK) CH_3_C(O)CH_2_CH_3_, has a flash point of −9 °C. There is also some water content. These “impurities” do not significantly change the flash point or the ignition properties of the liquid or vapor, but may give some luminosity to the flames compared to the pure alcohols. The main conclusion is that these liquids produce highly flammable vapors at normal room temperatures.

Dividing the stoichiometric concentrations for methanol and ethanol, i.e., 12.29% and 6.54%, by the LEL concentrations, i.e., 6.7% and 3.3%, gives 1.83 and 1.98, respectively. These values are marked in Figure 1 and it is seen that the stoichiometric compositions, i.e., where the combustion is most severe, corresponds to normal room temperatures. 

The molar mass of a mixture of air dry air and methanol at e.g., C/LEL = 2, i.e., 13.4%, is 0.02938, which is 1.4% more than dry air. The molar mass of a mixture of air dry air and ethanol at e.g., C/LEL = 2, i.e., 6.6%, is 0.03010 which is 3.9% more than dry air. This means that these gas-air mixtures are a few % heavier than the air and may therefore drain from a container and create a combustible atmosphere close to the unit being refilled. 

### 2.4. Thermal Expansion When Ignited, Density and Fluid Dynamics

In Equations (1) and (2), the number of gas molecules after combustion to the number of gas molecules prior to combustion is close to unity. Ignoring this minor change in number of molecules, the resulting immediate volume after ignition may then, in accordance to the ideal gas law, be estimated by the temperature change:

(5) $$V_{f}\approx V_{o}\frac{T_{f}}{T_{o}},$$

where $V_{o}$ (m^3^) is the flammable vapor volume at ambient temperature, and $T_{o}$ (K) and $T_{f}$ (K) are the resulting flame temperatures after combustion, i.e., before any heat losses take place. Usually, the LFL corresponds to an adiabatic limiting temperature of 1500–1600 K [31]. At room temperature of 23 °C (295 K), this corresponds to a 5–6 fold volume expansion. 

**Figure 3:**
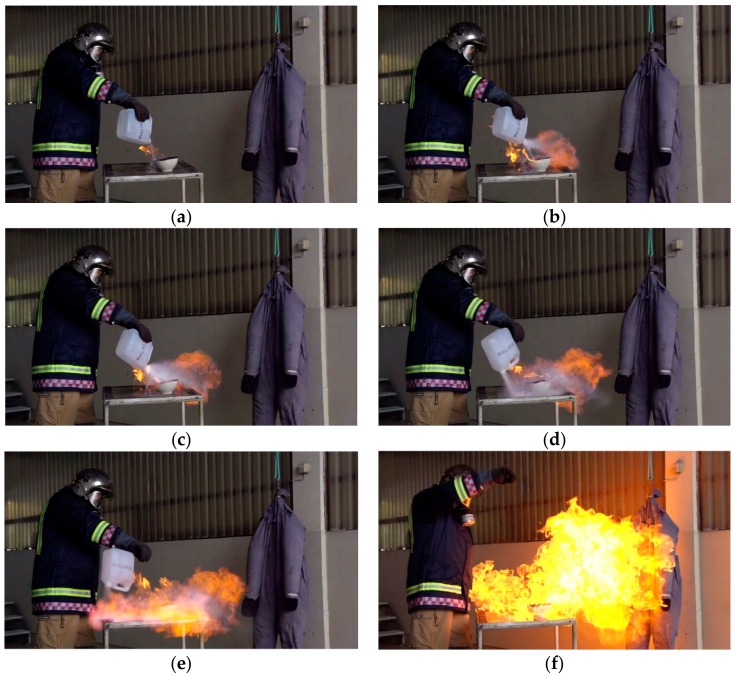
Demonstration of a 4 L ethanol container gas phase igniting at room temperature. The time sequence is about 0.5 s, i.e. (**a**) *t* = 0.1 s, (**b**) *t* = 0.2 s, (**c**) *t* = 0.3 s, (**d**) *t* = 0.4 s, (**e**) *t* = 0.5 s, (**f**) *t* = 0.6 s. (The Norwegian Directorate for Civil Protection (*DSB*) [32], reproduced with permission.).

Assuming now that a half-full methanol or ethanol fuel container has been kept at a this temperature, the gas air mixture inside the container will soon approach a concentration close to C/LFL ≈ 2, i.e., well within the flammable region and close to the stoichiometric worst case conditions. If liquid is then poured onto an ignition source, e.g., a burning flame, the poured heavy gas mixture and the liquid will catch fire. It is highly probable that the flame will propagate into the container and ignite the internal combustible air fuel gas mixture. As the internal gas volume ignites, the resulting volume expansion displaces the liquid in the bottom of the container which is then violently released through the container opening. The reaction force is acting below the hands of the person pouring liquid. The container will then be accelerated and rotate around the horizontal axis releasing even more burning liquid. Flames and burning liquid propelled out from the container may expose persons in the vicinity and inflict severe burns. This sequence, as demonstrated by a fire fighter, is shown in Figure 3. A video showing a chemical tornado in a real-life demonstration going wrong at a museum in Reno, Nevada, injuring 13 children, reveals the hazards involved [30].

It should be noted that also with bottle shaped containers, the reaction force usually works below the hand wrist. Given a similar ignition as demonstrated in Figure 3, it is most likely that a 1-L bottle will release its content in a similar way, i.e., propelling the liquid fuel several meters and/or rotating onto the person holding the bottle. The ignition is fast and the resulting reaction forces are probably so strong that it is not possible for an unaware person to compensate the involved reaction forces. Victims exposed to this danger neither have time, nor possibility, to direct the dangerous burning liquid in a safe direction. 

### 2.5. The Fire Plume Behavior

While a flame as shown in Figure 3f develops, it is generally known that the hot flames and combustion gases will rise as a result of buoyancy forces due to their low density. Since the average molar mass of the gas mixture does not change much during the combustion, i.e., nitrogen is the dominating gas species, the relative change in density may be estimated by the temperature change. Based on Equation (1), the density after combustion may be calculated by:

(6) $$\rho_{c}\approx \rho_{o}\frac{T_{o}}{T_{f}},$$

where $T_{o}$ and $T_{f}$ are the ambient temperature and the flame temperature, respectively. The hot flames of low density start rising relative to the ambient cold and heavy air. The rising hot gas plume is associated with lower internal pressures resulting in air entrainment [33]. If, however, there is a solid object, i.e., a person, close to this rising plume, the plume cannot entrain the object and is instead pulling itself toward the object, i.e., toward the person [31]. A person in the proximity to an ignited gas cloud at low levels may therefore tend to cling to a person while the flames rise, resulting in extended heat exposure. 

### 2.6. Burning Characteristics and Heat Flux to Exposed Objects

Liquid fuels, such as propane, butane, naphtha, petrol, kerosene, etc. all burn with a luminous flame resulting from glowing soot particles. Pure methanol and ethanol generally burn cleanly with a bluish flame color and very limited luminosity. In daylight, it may be difficult to spot such flames. Since water has a lower vapor pressure than methanol and ethanol, the liquid left in the burner unit will be enriched in water especially during the terminal phase of the combustion. This may result in very small and invisible flames during the last minutes before flame out. It may therefore be anticipated that tiny flames may persist in a seemingly extinguished bioethanol heater or fire place. The user may be unaware of these flames representing an ignition source during refueling.

The heat flux to an object exposed to hot flames may be expressed by:

(7) $${\dot{Q}}^{”}=h\left(T_{f}-T_{s}\right)+\phi \varepsilon_{f}\sigma T_{f}^{4}\;(W/m^{2}),$$

where h (W/m K) is the convective heat transfer coefficient, $T_{f}$ (K) is the temperature of the flame, $T_{s}$ (K) is the temperature of the exposed surface, ϕ is the view factor, ε is the flame emissivity, and σ (5.67 × 10^−8^ W/m^2^ K^4^) is the Stefan–Boltzmann constant. The emissivity of the flames is given by:

(8) $$\varepsilon_{f}=1-\exp\left(-KL\right),$$

where K (1/m) is the extinction coefficient and L (m) is the optical flame thickness. 

Methanol and ethanol burns very clean and is associated with very low extinction coefficients, typically about 0.37 [31]. This is an order of magnitude less than for other hydrocarbons. For small flame thicknesses, i.e., less than a foot, the emissivity as given by Equation (8) will be low and the heat transfer (Equation (7)) will be dominated by convection. Due to the lower radiant heat losses, i.e., low emissivity, the flame temperature is generally higher for the clean burning methanol and ethanol than for other hydrocarbons. Estimating the heat transfer coefficient may be difficult. However, the range of 20–30 W/m^2^K may be appropriate for this type of natural convection [34]. Assuming a flame temperature of 1500 K and a skin temperature of 310 K, this typically results in a convective heat flux of ≈30 kW/m^2^. Exposing naked skin to this heat flux quickly heats the skin surface, and the basal layer, to temperatures associated with burn injuries [35]. If combustible clothing textiles are exposed to flames of this heat flux or hit by burning liquid, the fabric is pilot-ignited almost instantaneously. Burning liquid in direct contact with the body may prolong the period of high heat fluxes and result in very severe burns.

Clean burning, with slightly bluish low-emissivity methanol and ethanol flames, makes small flames appear quite invisible (especially in daylight), representing a very characteristic threat. Being generally accustomed to luminous yellow and red flames, the users may be unaware of this invisible ignition source, which is characteristic of the ethanol and methanol flames.

## 3. Analysis of Selected Cases Described in the Literature

Several researchers have reported on skin burns as a result of accidents involving biofuel heaters [22,23,24,25,26,27]. During the literature study, some similar incidents were also found at educational demonstrations [29,30] and in dentist office treatments [28]. These involved methanol, which is in many ways a quite similar type of fuel, resulting in fire exposure and severe burns. Since these incidents shed light on similar risks as biofuel heater and fireplace accidents, they are included in the analysis.

### 3.1. The Selected Biofuel Burn Case Studies

The study by Kraemer et al. [22] presents two cases of patients presented to their intensive care unit in Germany succeeding burns related to bioethanol fuel. The first patient was a 45-year-old female with 30% total body surface area (TBSA) deep partial- and full-thickness flame burns involving the face, neck, right chest, thighs, and both arms and hands. The accident took place when the patient should refill a still burning design fireplace with bioethanol. A flash fire then occurred and the flame instantly set the patient and her clothing on fire. The patient was admitted for 14 days to their intensive care unit. The duration of the in-hospital stay until discharge was 4 weeks [22]. This burn accident clearly demonstrates the dangers of trying to refill the bioethanol fireplace while it is still burning. It is, however, not clear whether this incident involved ignition of the vapor phase in the refilling bottle.

The second case described by Kraemer et al. [22] involved a 46-year-old female patient who also prepared to refill her fireplace with bioethanol while it was still burning. With the bottle lid removed, and the bottle in her hand, she tripped and sprayed liquid onto the burning fireplace. A flash fire followed, and resulted in an explosion of the internal bottle gas volume, exposing the patient to the fire. This patient suffered partial-thickness burns to the face, left arm, both hands, and the entire right leg, affecting a TBSA of 12%. The patient was admitted to their intensive care unit for 4 days. The duration of the in-hospital stay until discharge was 2 weeks [22]. This burn accident also demonstrates the dangers of approaching the fireplace for refilling while it was still burning. In this case it was also evident that the container vapor phase ignition contributed to the severity of the accident.

A study by Heald and Muller [24] reported on five burn victims in Australia in 2014. All incidents involved biofuel heaters and occurred while refueling the units. The first incident involved refueling a table top heater which was believed to have been extinguished for 20–30 min. During refilling, a large fireball and explosion occurred, which exposed two patients sitting opposite the table top heater. The first patient, a 28-year-old male, sustained 43% TBSA deep partial- and full-thickness burns to the face, neck, anterior and posterior trunk, arms, legs and left foot. The patient was in the intensive care unit for 26 days and was discharged from hospital after 57 days. A 22-year-old female was involved in the same incident. She sustained 31% TBSA deep partial- and full-thickness burns to the face, neck, anterior and posterior trunk, arms, and left leg. She was discharged from hospital after 78 days. This incident clearly demonstrates the dangers related to refueling a burning unit and clearly involved container vapor phase ignition and explosion. 

**Figure 4:**
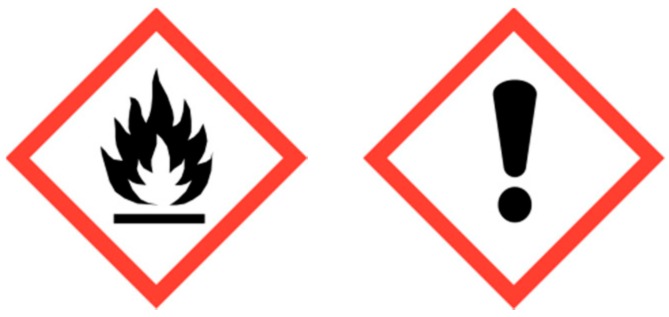
Hazard communication pictograms for bioethanol (**left** fire risk, **right** health risk).

The third patient described by Heald and Muller [24] experienced an explosion while refilling an outdoor biofuel heater. It was unclear whether the flame was extinguished or not, or whether the unit was still warm. The burn victim suffered 45.5% TBSA deep partial- and full-thickness burns to the face, neck, trunk, arms, hands, and legs and was discharged from hospital after 63 days. The fourth patient described by Heald and Muller [24] was refilling an indoor coffee table biofuel heater which, according to the patient, had been extinguished for at least 40 min. The fuel was methylated spirit, and when refilling, he and the fuel container he was holding were engulfed in flames. This burn victim suffered 10% TBSA superficial and deep partial-thickness burns to the face, neck, right arm and hand, and right leg. He was discharged after 6 days hospital admission. The fifth patient described by Heald and Muller [24] was sitting at an outdoor table 1.5 m away from a table top heater to be refilled after being extinguished for nearly 20 min. When the reservoir was refilled, a large fireball engulfed her. This burn victim suffered 4% TBSA superficial and deep partial-thickness burns to the face, neck, and left hand. She was discharged from hospital after 5 days. It is evident that for all these last three patients, the container vapor phase was involved in the combustion process. The fireballs described cannot be explained by the 5–6 fold gas phase expansion during combustion, i.e., expelled liquid must have been involved, as shown in Figure 4.

The study by Jaehn et al. [27] presents a case study regarding a 42-year-old female suffering severe burns after improper handling of liquid bioethanol. The patient suffered burns to 55% of the TBSA; 28% were deep partial- and full-thickness burns. The patient was in the intensive care unit for 7 weeks, with a 2-month hospital stay followed by several weeks of rehabilitation treatment for heavy burn injuries.

### 3.2. The Selected Biofuel Group Studies

The study by Van Zoonen et al. [23] presents a summary of two burn victims in the Netherlands in 2010 and 29 burn victims in 2011. First, three experts on bioethanol burners were questioned for framing non steering semi-instructed interviews of 14 bioethanol burn victims. They found that for eight of these incidents, bioethanol was misused as an accelerant for lighting a fire or a barbeque, and not for the intended use, i.e., bioethanol burners. They also found that all the interviewees had poor knowledge about the fuel and conditions of use. Many of the bioethanol bottles were half-full and internal gas phase explosion was concluded as a major contributor to the severe outcomes. In all the cases, flames were present when adding new bioethanol fuel. Van Zoonen et al. [23] concluded that the inexpensive and easily obtainable product may have created an image of an innocent product rather than a product that might result in severe burn incidents. This series of burn incidents clearly demonstrates the risks associated with the application of bioethanol when there is a flame in place. It was also evident that vapor phase ignition contributed to the severity of several of the accidents.

The study by Neubrech et al. [25] analyzed 12 patients (7 males and 5 females, 19 to 53 years of age) suffering bioethanol-associated burn injuries in Ludwigshafen, Germany, in the period 2010–2014. The incidents resulted in burns to an average of 17% (±9% SD) of the TBSA, commonly involving the face and the hands. The median length of hospitalization was 20 days (range 3 to 121). Intensive care unit treatment was required 1 to 64 days (median 4.5 days). In most cases, these injuries took place while starting the fire (3) or refilling (7), and happened even though the safety instructions were followed. The authors concluded that bioethanol-fueled fireplaces for home decoration represent potential sources for severe burns even when used as intended [25]. This study clearly demonstrates the dangers of both firing and refilling of the biofuel units. There are no details regarding involvement of the container or bottle vapor phase, but that is quite likely, at least for the most severe incidents.

### 3.3. The Selected Methanol Burn Incidents

The U.S. Chemical Safety and Hazard Investigation Board (CSB) usually engages in major industrial accidents. In special circumstances it may, however, also engage in civil sector incidents. For example, two accidents related to demonstrations for children (one at a school and one at a museum) involved methanol, and caught the attention of the CSB, who issued a safety bulletin for key lessons [30]. In both these cases, a burning flame ignited the vapor phase of a methanol container during refilling. In the first incident, an internal container gas phase explosion propelled burning methanol and caused burns to children at a school chemistry demonstration. In the second incident, a similar situation resulted in burns for 13 children during a chemical demonstration at a museum. The CSB stressed several safety measures in order to prevent similar incidents in the future [30]. A cellular phone video clearly demonstrates the severity of the accident [29]. Both incidents involved an internal container gas volume explosion propelling burning liquid at the children. 

The study by Sohal et al. [28] presents a case study of a rare burn mishap involving a 46-year-old female during endodontic treatment. The incident happened during obturation with Gutta–Percha (GP). Whilst condensing the GP, the assistant was instructed to add methylated spirit (methanol) into the gallipot to keep the flame burning. The gallipot burner was about 2 feet from the patient while the methanol was added. An explosion knocked the gallipot burner onto the patient, inflicting burns to the right cheek, right lateral neck, and on the upper right arm, causing a second-degree burn injury covering 9% of the TBSA. She was discharged from the hospital after two weeks with minimum esthetic impairment. In order to get the explosion force moving the galipot as described in this case, the internal bottle gas volume must have been involved in the combustion process. 

### 3.4. Summary of the Studied Burn Injuries

In most of the cases studied, ignition of the container gas phase was an important part of the accident mechanism. This may happen only when there is an ignition source, which in most, if not all, cases were a persistent burning flame. Such a flame may, as explained in Section 2, be quite invisible, especially when close to the point of extinction. Some few cases were, however, also related to the first time the unit was set alight [25]. These may be due to heavy methanol gas being spilled from the container and ignited when the intention was to ignite the burner. 

Guillaume et al. [26] highlight two families of risks related to biofuel heaters, i.e., heat injuries during use or refill and the effect on air quality. They recorded heat release rate and tank temperatures during use as well as after extinction. It was noted that the highest tank temperatures, reaching above 100 °C, were recorded well after the flames had died out. 

Since ethanol has higher molecular mass than air, 46.07 g/mol versus 28.97 g/mol, the ethanol vapor produced will, regardless of being warmer than the ambient air, be heavy and therefore not rise buoyantly. Computational fluid dynamics (CFD) modeling was used by Guillaume et al. [26] to model the flammable vapor cloud during refilling, clearly demonstrating that for the particular unit investigated, a person refilling the unit was at severe risk of burns if refilling and reigniting the unit when still warm. If ignited, the produced vapor cloud may severely burn the person operating the unit. There is also a risk that he/she will not remove the refilling container, which may be involved in a fire scenario.

## 4. Possible Risk Reducing Measures

### 4.1. Heat Fluxes Associated with Burning Liquid on Fabric

In general, there is very limited research available regarding spills of flammable liquids on clothing regarding spilled methanol or ethanol. This has, however, been studied for spills on carpets by Ma et al. [36]. Studies of spills on vertical fabric and heat fluxes to a body mannequin could reveal important information about the heat transfer and possible preventive measures such as prompt removal of the soaked fabric, etc. 

### 4.2. General Safety Education

Professionals may properly understand hazard pictograms for dangerous chemicals. However, based on the standard hazard pictograms for bioethanol (and methanol), as depicted in Figure 4, lay people may probably not understand that the liquid under quite normal circumstances represents an explosion threat. 

General safety courses with emphasis on flammable liquids may help preventing future accidents. In such teaching, one may possibly include videos showing real accidents, such as the one in Reno, Nevada, resulting in 13 children with burn injuries [29]. Video demonstrations, such as the one shared by The Norwegian National Directorate of Civil Protection [32], may also be valuable. It is generally recommended that such videos should be backed up by scientific explanations tailored to the audience at hand. 

As the units considered in the present study are used by nonprofessionals, it may, however, be difficult to reach out to the new customers as well as ensuring that the risk is still understood and remembered when using the unit several years later. A search for robust technical barriers should be compulsory.

### 4.3. Possible Technical Barriers

A good safety measure would be to prevent ignition of gas or liquid during refilling operations. There may be several ways of achieving this. Some bio heater and fireplace units are equipped with a thermostat giving a sound when sufficiently cold to be refueled. However, the unit may not burn as intended if put on a sloping surface or when distorted/buckled by previous heat exposure. Small flames may then persist in parts of the unit, i.e., not heating the thermostat. The thermostat may therefore not be recognized as robust barrier [26].

It is well known in the safety community that flames cannot travel through small openings. The minimum distance, which is called minimum experimental safe gap (MESG), is dependent on the gas type and the gas air ratio. The MESG is smallest for concentrations of gas slightly richer than the stoichiometric ratio. For methanol and ethanol, the MESG is 0.92 mm and 0.89 mm, respectively [37]. 

Equipping the refilling bottle or container with a strainer with openings smaller than the MESG would prevent the propagation of an external flame into the container gas phase. Prolonged heating of a strainer may, at least in the theory, result in flame propagation through the strainer. A deeper slid, e.g., X-shaped, to support fluid flow out of the container and air ingress into the container, may be a safer alternative. This would also limit the flow of heavy gas out of the container while filling or refilling fuel. This technical barrier could indeed limit most of the severe accidents related to these biofuel heaters studied in the present work.

### 4.4. Quickly Cooling Heat Exposed Skin

If the skin has been exposed to flames or burning liquid, it is well known among safety and health worker professionals that prompt cooling of the skin by tempered water may limit the burn consequences significantly [8,14,34,38,39]. Cooling the burned skin with tempered water is generally beneficial for up to 20 min, while cooling of the patient as such should be avoided [39]. The best way to achieve prompt cooling would be to strengthen the general first aid education by including prompt cooling of any overheated skin, e.g., flame exposure, liquid scalding, food scalding, contact with hot objects, etc.

## 5. Discussion

In the present study, the physical and chemical characteristics of methanol and ethanol (bioethanol) have been outlined. It has been demonstrated that the vapor pressures of these liquids at normal room temperatures create highly combustible gas-air mixtures when in equilibrium with the liquid phase. At 22–25 °C, the fuel-to-air ratio is very close to the stoichiometric composition. This is also the most easily ignited composition, either by static electricity or an open flame, and results in the worst case scenarios regarding explosion strength. 

The ethanol-air mixture is heavier than air and may pour out of a container and result in a combustible gas air pillow which may ignite when trying to light a biofuel fireplace or heater unit. The gas is invisible, and the operator may probably not be aware of this situation. It has been shown that the container or bottle gas phase may likely become ignited when refilling a biofuel fireplace or biofuel heater which is still burning. 

It has been outlined why such fuels may burn with a flame that is quite invisible in day light, especially the last period before the flame self-extinguishes. This persistent flame, thought by the user to be extinguished, represents a very dangerous ignition source, especially when refilling the unit. It may quite likely ignite the vapors being poured out of the refilling container or bottle. During this process, it is quite likely that explosive container gas volume will be ignited, resulting in violent explosion propelling burning liquid out of the container or bottle. The operator, as well as people several meters away, may then be engulfed in flames and soaked with burning liquid exposing naked skin or setting the clothing fabric on fire. 

A number of severe bioethanol burn incidents are presented in the literature [22,24,26,27,28] and two group studies [23,25] were analyzed based on the outlined possible accident mechanisms. Three cases involving very similar, and severe burn, incidents with methanol were also included [28,29,30]. Two of the methanol burn incidents injured several children at science demonstrations. For all these burn incidents, it turned out that the most severe accidents involved ignition of the container gas phase resulting in a violent explosion expelling burning liquid from the container. Since the auto ignition temperatures (AITs) of methanol and ethanol are quite high, i.e., respectively 470 °C and 365 °C, respectively, it is not likely that hot surfaces without a flame represented the ignition source. A persistent flame, unnoticed by the operator, was likely the ignition source in most, if not all, of these incidents. A minority of the incidents represented ignition of the vapor cloud that had been poured from the liquid container or bottle without involving the container or the bottle. 

Based on the knowledge that flames cannot travel though small openings, it is suggested to develop bottles and containers with openings less than the maximum experimental safe gap (MESG). Openings less than about 0.9 mm would ensure that no flame could propagate into the container to involve the internal gas phase. Such a restriction may also limit the amount of heavy gas air mixture being poured out of the container while filling or refilling a bio heater or fireplace unit. It is suggested that research is performed on this issue to develop safer containers and bottles, and safer refilling possibilities preventing extensive evaporation if refilling a still warm unit.

Van Zoonen et al. [23] discovered through interviews with burn victims that they a poor understanding of the hazards involved. The fuel is easily available to a low cost and it does have an odor associated with typically highly flammable long-chained non-oxidized hydrocarbons, e.g., gasoline or naphtha. In may therefore not be associated with any recognizable danger. In order to mitigate this, safety courses may be updated with videos [29,32] and information brochures explaining the risks associated with bioethanol and methanol. It is also necessary to focus on mitigating measures, such as prompt and cooling if a burn incident has happened. In order to develop efficient safety courses, it is therefore recommended to analyze the present level of risk understanding in the public in order to find at which level the teaching should start. 

The heat exposure may also be a result of hot liquid scalding in combination with flame exposure, which has been expensively researched for hot beverage and hot food spills. The injury mechanisms may therefore be quite complicated as both flame exposure and hot liquid scalding due to wetted fabric contribute to the resulting heat injury. Promptly removing clothing is therefore vital to get the overheated skin cooled by tempered water [8,34,39,40,41].

The bottles and containers are marked as flammable and with a health risk. The pictograms indicating the hazards of methanol and bioethanol fuels do not, however, give any indication of an explosion risk. This risk may be understood by professionals. It may, however, be questioned whether communicating flammability and health risk is sufficient for the public to gain the proper understanding of the risks involved when handling these liquids. 

Recently, infrared (IR) cameras have been developed for finding gas leakages in e.g., the oil and gas industry [42]. For future research on methanol and ethanol burn incidents, such equipment, which detects the C-H bonds in the methane and long-chained hydrocarbon molecules, may be tested for methanol and ethanol. It is quite likely that doing research with this equipment may reveal and make visible some of the mechanisms outlined in the present work. It is suggested that such research be initiated to gain understanding as well as to produce material for better public safety courses.

It is important to be aware of accidents related to dislocation of the units, i.e., units detaching from wall mounts, being interfered with, or falling from tables. These fireplaces/heaters can be dangerous for children who may experience large-TBSA burns even when exposed to comparably small fires.

## 6. Conclusions

In the present study, the possible mechanisms leading to accidental fires involving methanol and ethanol/bioethanol liquid and vapor phase have been outlined. Burn accidents previously described in the literature have been analyzed based on the outlined accident mechanisms. It turns out that equilibrium vapor pressure close to the stoichiometric fuel–air composition represents a very severe risk when handling these liquids. Some accidents have been related to the ethanol/bioethanol fuel–air mixture being poured out of the container, resulting in a combustible cloud accumulating close to the unit to be filled. Similar clouds have also been accumulated when refilling a warm unit. When approaching the unit for ignition, this vapor cloud ignites and engulfs the operator in flames, typically on the chest, face, and arms. 

Ignition of the vapor phase resulting in an internal explosion expelling burning liquid resulted in the most of the accidents and generally resulted in the most severe burns. Not only the operators but also people several meters away were exposed to flames and soaked in burning fuel. The ignition source was in most, if not all these burn incidents, most likely a small invisible flame from previous use of the unit.

By understanding the mechanisms involved and analyzing accidents, several risk-reducing measures are suggested for further research in order to reduce this very severe burn risk.
